# Effect of rapid maxillary expansion on the apnoea-hypopnoea index during sleep in children. Systematic review

**DOI:** 10.4317/jced.59750

**Published:** 2022-09-01

**Authors:** Eva Martos-Cobo, Pedro Mayoral-Sanz, Antonio-Javier Expósito-Delgado, Joaquín Durán-Cantolla

**Affiliations:** 1 Máster en Ortodoncia Avanzada Universidad Europea de Madrid. Máster de Trastornos del Sueño. Universidad País Vasco. Práctica privada Clínica Olavide Jaén; 2 Máster en Medicina Dental del Sueño, Universidad Católica de Murcia UCAM; 3 Coordinador Prestación Dental Infantil. Hospital Universitario de Jáen; 4 Instituto de Investigación, OSI Araba, Hospital Universitario de Araba, Vitoria-Gasteiz, Álava, España

## Abstract

**Background:**

Rapid maxillary expansion (RME) treatment is prescribed in patients with maxillary compression, achieving increases in transverse palate and nasal cavity dimensions together with an increase in the distance between the pterygoid processes. Sleep apnoea-hypopnoea syndrome (SAHS) in children is often associated with anatomical risk factors and treatment may involve surgery, drugs, dentofacial orthopaedics, myofunctional and positional approaches.

**Material and Methods:**

The aim of this systematic review it to obtain scientific evidence of the effect of RME on the apnoea-hypopnoea index (AHI) in growing patients. PubMed, Cochrane Library and EMBASE were the online databases used for the search. The scientific publications selected met the following inclusion criteria: articles published from 2011 to May 2021; growing patients undergoing rapid maxillary expansion surgery; and studies with records of AHI before and after rapid maxillary expansion using polysomnography or respiratory polygraphy.

**Results:**

Seven articles that provided the necessary quality of scientific evidence were finally selected. The review followed the Cochrane Handbook for Systematic Reviews of Interventions, version 5.1.0, and the GRADE approach for rating the certainty of evidence. Data analysis was performed using Numbers 4.3 and ReviewManager (RevMan) 5.4.1 software and GRADEpro and Mendeley online platforms.

**Conclusions:**

The results show a reduction in AHI following RME therapy in growing patients. More research is needed with larger sample sizes, more specific inclusion criteria and standardised data sharing.

** Key words:**Rapid maxillary expansion, maxillary distraction, sleep apnoea, children.

## Introduction

Breathing is one of our body’s vital functions. Various factors may affect a person’s breathing. Adenotonsillar hypertrophy, obesity, craniofacial abnormalities and mandibular hypoplasia or retrognathia are just some of the factors that can affect breathing. The size of the airway is conditioned by the dysmorphism, resulting in dysfunction (1), and therefore we must retain or restore adequate breathing function.

Sleep apnoea-hypopnoea syndrome encompasses two different types of respiratory problems: central (originating in the central nervous system), obstructive (collapse of the anatomical structures of the upper airway) and mixed (central and obstructive clinical signs at the same time).

Apnoea is considered when there is a reduction in airflow of at least 90% that lasts for at least two breathing cycles. The term hypopnoea is used when the reduction in airflow is 30-90% and it is associated with an arousal or a >3% oxygen desaturation. Specifically for this population group, diagnostic criteria include snoring, changes in heart rate and paradoxical breathing (2,3).

The pathophysiology of total or partial obstruction of the upper airway is based on two processes: intermittent hypoxia and sleep fragmentation. Recurrent episodes may then trigger other processes: oxidative stress, increased sympathetic activity, increased blood pressure, vasoconstriction or endothelial dysfunction.

SAHS leads to a pro-inflammatory state, early diastolic dysfunction and arterial hypertension (4), failure to thrive, metabolic changes (5), attention deficit, hyperactivity (6), daytime sleepiness, cognitive impairment (7) and emotional lability. Magnetic resonance imaging has even detected decreased sensitivity to harm in the left amygdala (8), which reduces patient empathy. With regards to oral health, there is a direct relationship between tooth wear and the severity of SAHS (9). Risk factors that affect SAHS include adenotonsillar hypertrophy and obesity (10). There are some authors who describe subgroups of SAHS. Gozal subdivides cases into type I for those cases of SAHS with adenotonsillar hypertrophy and type II for cases of SAHS associated with obesity (11).

Sleep apnoea-hypopnoea syndrome in children is associated with processes that have a negative impact on the patient’s health and growth-development. Maxillary compression may increase resistance to nasal airflow, creating a mouth breathing habit. The tongue changes position and is often pressed against the lower incisors, thereby changing mandibular position and growth. A feedback loop is created, making nasal breathing more and more difficult. It is estimated that SAHS affects around 2-3% of this population. Early diagnosis of SAHS and personalised treatment will have a positive impact on the child’s overall health, growth and development and a positive socio-economic impact. Paediatric SAHS can be treated using a variety of approaches: surgery, drugs, dentofacial orthopaedics and muscle rehabilitation. Rapid maxillary expansion helps modify the anatomical structures involved, including lip seal, and makes it easier to resume normal breathing (12-21). Achieving more normal breathing and more favourable anatomical structures may reduce the AHI.

Rapid maxillary expansion is a therapeutic procedure based on palatine bone distraction. The amount of expansion required is calculated during the treatment planning stage. The orthopaedic device is attached to the actual teeth or palatine bones using skeletal anchorages and the appropriate activation rate is determined. The activation frequency varies according to the expansion protocol but it generally ranges from 0.25 to 0.5 mm per day. The goal of RME treatment is to separate the two palatine bones and to hold them in this new position until the midpalatal suture partially ossifies (12).

During the active phase, treatment on the lower arch can cause clockwise rotation of the mandible due to the excess bite-opening effect of the orthopaedic device. Consequently, expanders should be designed according to each patient’s anatomy to avoid backward rotation of the mandible (13) On removing the expander, the mandible rotates anti-clockwise and may return to an even more anterior position in the sagittal plane due to the fact that the limiting and compressive effect of the narrow upper arch has been reduced.

Effects include an increase in transverse dental arch dimensions and a small decrease in dental arch depth. The maxillary interincisal space acutely increases during the active phase. However, this new diastema tends to shrink again due to the effect of the transseptal fibres of the periodontal membrane. If the design of the intraoral device is not appropriate, the curve of Wilson (which joins the cusp tips of the molars in the transverse plane) may increase. A disturbance in the curve of Wilson may create an occlusal interference due to the palatal cusps tilting and therefore great care should be taken when designing and fabricating the expander (14). With regards to soft tissue, RME treatment slightly decreases tongue length and vertical airway dimensions, increases philtrum and nasal base widths and columella height and also causes a small increase in oropharyngeal width (15,16). The hyoid bone is displaced upward (15).

The treatment of SAHS in children is primarily surgical (12,22,23). Adenotonsillar hypertrophy is the main risk factor and has a high prevalence in children (24). Therefore, adenotonsillectomy or drug treatment (25,26) for such hypertrophy have until now been the first-line treatments for SAHS in this population group in preference to facial orthopaedics, myofunctional therapy and positional control (27-29). Childhood mortality due to adenotonsillectomy may be prevented in many cases in which RME is prescribed to treat SAHS.

Deficient research into SAHS in children and a lack of consensus between scientific evidence and action protocols among professionals justify this systematic review, the objective of which is to analyse the effect of rapid maxillary expansion on the apnoea-hypopnoea index in children.

## Material and Methods

A protocol was drafted during the review design and planning phase in compliance with the Cochrane Handbook for Systematic Reviews of Interventions, version 5.1.0.29 The protocol included the articles’ inclusion criteria, study search and identification methods and data collection and analysis methods. The protocol included summary of findings and assessment of the certainty of the evidence forms.

The study design took the PRISMA Statement30 into consideration. The following question was put forward: Can rapid maxillary expansion versus no interventions affect the paediatric patient’s AHI? The corresponding PICO elements are:

Population: non-syndromic growing patients requiring maxillary expansion.

Interventions: Rapid maxillary expansion and recording of AHI.

Comparisons: No interventions and pre-treatment AHI.

Outcomes: AHI and O2 saturation.

The inclusion criteria were clinical articles published from 2011 to May 2021 in growing patients up to 16 years of age treated with rapid maxillary expansion surgery and with records of AHI before and after RME measured using polysomnography or respiratory polygraphy. With regards to exclusion criteria, no studies involving surgically-assisted rapid maxillary expansion or rapid maxillary expansion in combination with maxillary surgery were included.

The PubMed, Cochrane Library and EMBASE online databases were used for the study search. The review followed the Cochrane Handbook for Systematic Reviews of Interventions, version 5.1.0, and the GRADE approach for rating the certainty of evidence. The keywords used for the search were: rapid maxillary expansion, maxillary distraction, sleep apnea, children. A string of terms was used for the search with the Boolean operator “AND”. The search strategy was: “sleep apnea AND maxillary expansion”, “sleep apnea AND rapid maxillary expansion”, “sleep apnea AND maxillary distraction”, “children AND maxillary expansion AND sleep apnea”.

The initial screening search was carried out on titles and abstracts. Articles meeting the inclusion criteria were then analysed. Data extracted from these articles were included in the Numbers 4.3 database. Two Tables of contents were created. The first listed the following information for each study: author, year, title, identifier, study type, sample size, sample age, method, results and conclusion; and the second Table listed: author and year, sample size, age, deviation of ages, AHI prior to RME treatment, deviation of AHI prior to RME treatment, AHI after RME treatment, deviation of AHI after RME treatment, oxygen saturation prior to RME treatment, deviation of oxygen saturation prior to RME treatment, oxygen saturation after RME treatment, deviation of oxygen saturation after RME treatment.

The risk-of-bias assessment was conducted using ReviewManager (RevMan) 5.4. This assessment included the following domains: random sequence generation, allocation concealment, blinding of participants and personnel, blinding of outcome assessment, incomplete outcome data, selective reporting and other bias.

Mean differences of the treatment effect measures were recorded for continuous outcomes with a 95% confidence interval using ReviewManager (RevMan) 5.4.1. The data obtained for our study endpoints (AHI and SpO2) are shown in Tables 1-2.

The strategy for handling missing outcome data was split into two sequential processes: firstly contact with the author to complete the data; and second, when access to the author was not possible or data remained incomplete, an average was calculated for the missing values. In the 2015 study by Villa, post-treatment data were divided into subgroups of responders and non-responders. The average value was calculated based on the number of cases in each group.

The studies had different designs with regards to the application of individual treatment, either before or after surgery. There was also a variation in the length of time over which polysomnographic data were collected after RME treatment. For our study design, we decided to include patients who had received orthopaedic treatment and also had a polygraphic or polysomnographic record from before and after treatment. Subgroups that met the relevant inclusion criteria were selected, discarding data for patients who had undergone prior surgery and also control data at 24 months; this increased the homogeneity of the sample. Heterogeneity was measured by calculating I2.

The RME procedure requires that the patient wear an intraoral appliance, which must be checked periodically by the patient’s orthodontist. This treatment prevents the blinding of patients and personnel. The studies included in this review do not state whether they involved any blinding or not so it has been considered that there was no blinding for the risk-of-bias assessment.

Numbers 4.3 software was used to collate information for data synthesis while ReviewManager (RevMan) 5.4.1. was used for data analysis. The summary of findings is described in the Results section and shown in the Tables and plots. The GRADE system for rating the certainty of evidence was used via the GRADEPro platform to assess the certainty of evidence.

## Results

After completing the literature search, seven articles were selected (17,31-36). All of the articles are related to clinical trials. The 2011 study by Guilleminault is a randomised, controlled clinical trial. Only the results from first-phase group 2 were collected from this study. These data correspond to 14 patients who received only RME treatment during this phase without prior adenotonsillectomy.

The 2011 study by Villa observed the effect of RME on the AHI in children with measurements at 12 and 24 months. Follow-up values at 24 months were discarded and the AHI and SpO2 data corresponding to groups T0 and T1 (pre- and post-RME treatment) were used. The Pirelli study was published in 2013. This paper compares the effect on the AHI of adenotonsillectomy in group 2 with RME treatment in group 1. Only the data from group 1 were taken into account. The 2013 study by Caprioglio observed the effect of RME on the AHI after 12 months of treatment. In 2014, Villa published a study involving 3 groups. Group 1 included patients treated with adenotonsillectomy, group 2 included those treated with RME and group 3 included patients who received both treatments. Only the data from group 2 were used. In 2015, Villa published a study with follow-up after 10 years of RME treatment that observed the effect of RME on the AHI. The data from before RME treatment and the data after 12 months of orthopaedic treatment were used.

All of the studies measured the AHI using polysomnography, which was performed on patients prior to treatment and after 12 months of treatment; the only exception is the 2011 Guilleminault study, which does not specify the time between the two polygraphic readings.

**Figure 1:**
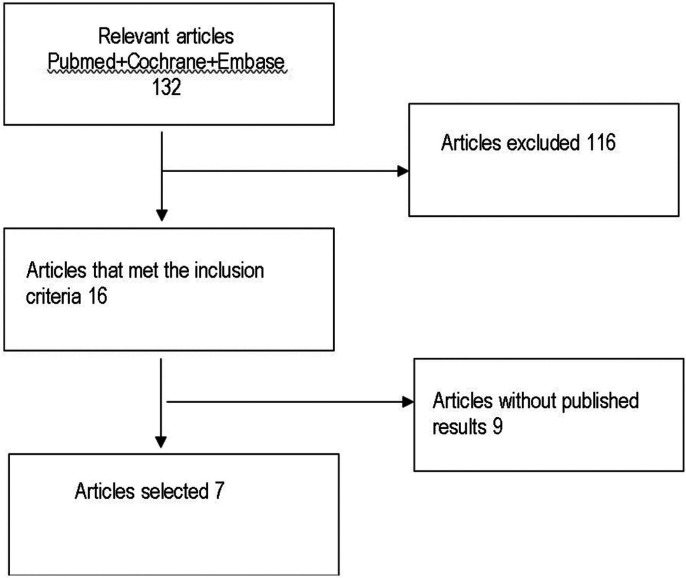
Article selection flow chart.

All studies, except for the 2012 Pirelli study, recorded oxygen saturation values (SpO2) before and after RME treatment at 12 months. The results of these selected articles describe a reduction in AHI and an increase in SpO2 after RME treatment. Mean differences in effect measures were recorded for continuous outcomes with a 95% confidence interval, obtaining the data and funnel plots shown in  Table 1, Table 2. The data correspond to the effect of RME treatment on the AHI and, as a secondary endpoint, on oxygen saturation (SpO2). The risk-of-bias assessment and the quality of evidence results are shown in  Table 3, Table 4 and Figure 1.

**Table 1:**
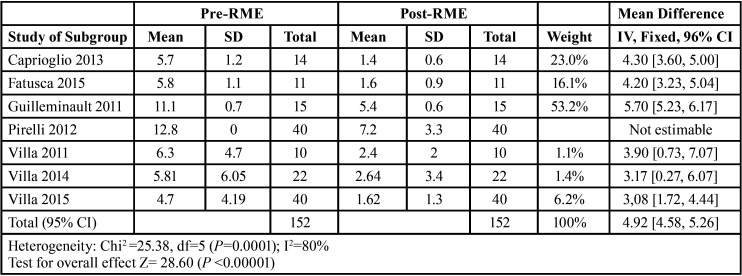
SpO2 results. Effect measures, I2, Z-test, weight of study.

**Table 2:**
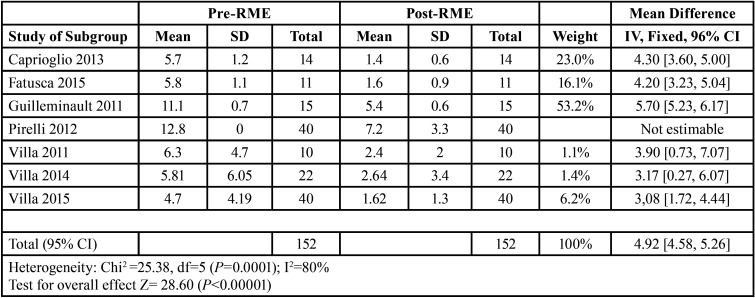
SpO2 results. Effect measures, I2, Z-test, weight of study.

**Table 3:**
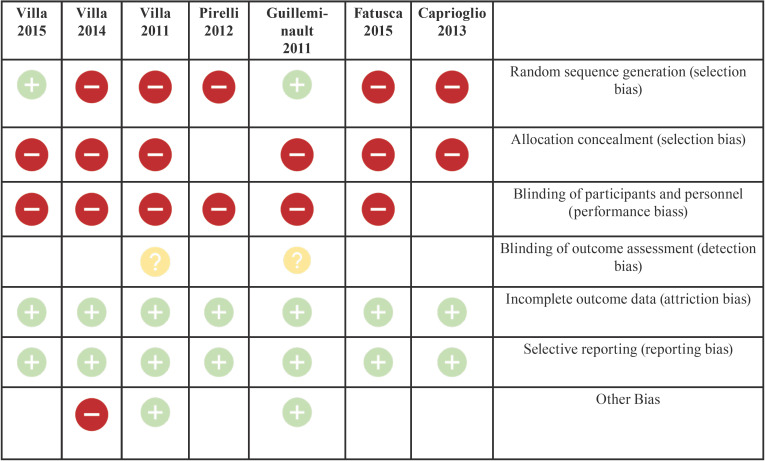
Risk-of-bias assessment with identification of article.

**Table 4:**
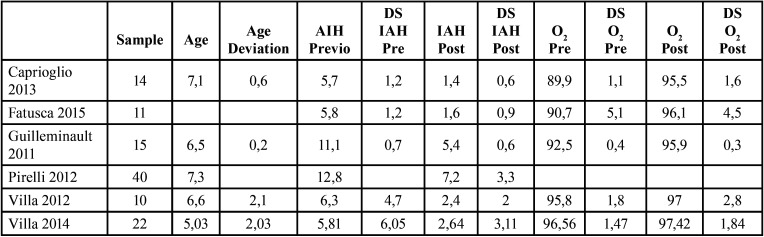
Summary of study effect data.

To select the sample, the design of the 2011 Guilleminault study only established a random sequence. The 2014 Villa study consecutively allocated study group participants. In all of the other studies, participants were selected based on their treatment needs: presence or absence of adenotonsillar hypertrophy, need for orthopaedic treatment and highest AHI. There is no information regarding allocation concealment, blinding of participants and personnel or blinding of assessors. Since RME is an invasive treatment requiring control and follow-up by professionals, it was assumed that neither allocation concealment nor blinding of patients and personnel were performed. We also have no information regarding the blinding of assessors and therefore an unclear risk was estimated. There were no incomplete data and outcomes were selectively reported.

Rapid maxillary expansion has a significant effect on reducing the AHI and increasing oxygen saturation. Individual data from each study are summarised by Number 4.3 software in Table 4. A full analysis of the effect measures for each study endpoint (AHI and SpO2) are shown in Tables 1-4. The most relevant study is the study conducted by Guilleminault in 2011 with 52.2% for the effect of RME on the AHI and 79.4% for the effect of RME on SpO2.

## Discussion

A decrease in the AHI following RME treatment was reported in the selected studies. Furthermore, oxygen saturation increased following said treatment. The certainty of the evidence assessed scored highly for the two effects studied.

Regarding possible bias during the review process, it should be noted that the protocol was drafted prior to the start of the literature search. However, it was not registered in PROSPERO, Cochrane or any other database for systematic reviews. Nevertheless, studies were found in Cochrane Library with designs that met the inclusion criteria but their results have not been published. They were therefore excluded from this review, despite their study design quality.

The practical conclusion reached is that rapid maxillary expansion is effective for decreasing the AHI and increasing blood oxygen saturation. Plans to perform SAHS treatment in this population group should therefore systematically include a facial-orthopaedic diagnosis that reduces the morbidity associated with other more invasive treatments.

Research should focus on the need to provide more evidence regarding this treatment. The design of the studies is heterogeneous and they consider different variables and measurements. For our study, those subgroups from each study that were not affected by other variables were selected. The data are still heterogeneous with an I2 of 80% and 95% for the two effects. Further research is required to clearly determine the effects caused by RME treatment.

The design of future studies should be more rigorous, and subgroups of prior (presence or absence of adenotonsillar hypertrophy, mouth breathing or tongue thrust habit) or induced variables (use of myofunctional therapy, drugs or adenoid-tonsil surgery) that improve the evidence of rapid maxillary expansion treatment in SAHS in children should be established.
